# Monitoring health disparities in healthcare-associated infection surveillance: A Society for Healthcare Epidemiology of America (SHEA) Research Network (SRN) Survey

**DOI:** 10.1017/ice.2023.181

**Published:** 2024-04

**Authors:** Caitlin L. McGrath, Latania K. Logan, Valerie M. Deloney, Lorry G. Rubin, Karen A. Ravin, Martha Muller, Allison H. Bartlett, Annabelle de St. Maurice, W. Matthew Linam, Carolyn Caughell, Lynn Ramirez-Avila

**Affiliations:** 1 Department of Pediatrics, University of Washington, Seattle, Washington; 2 Department of Pediatrics, Emory University School of Medicine, Atlanta, Georgia; 3 Children’s Healthcare of Atlanta, Atlanta, Georgia; 4 Society for Healthcare Epidemiology of America, Arlington, Virginia; 5 Department of Pediatrics, Cohen Children’s Medical Center, Northwell Health, New Hyde Park, New York; 6 Donald and Barbara Zucker School of Medicine at Hofstra/Northwell, Hempstead, New York; 7 Division of Infectious Diseases, Nemours Children’s Hospital Delaware, Wilmington, Delaware; 8 Department of Pediatrics, Sidney Kimmel Medical College at Thomas Jefferson University, Philadelphia, Pennsylvania; 9 Department of Pediatrics, Division of Infectious Diseases, University of New Mexico, Albuquerque, New Mexico; 10 Section of Pediatric Infectious Diseases, Department of Pediatrics, The University of Chicago Medicine Comer Children’s Hospital, Chicago, Illinois; 11 UCLA David Geffen School of Medicine, Los Angeles, California; 12 Hospital Epidemiology and Infection Prevention, Department of Quality, University of California San Francisco Health, San Francisco, California; 13 Division of Pediatric Infectious Diseases and Global Health, University of California–San Francisco, San Francisco, California

## Abstract

We investigated whether and how infection prevention programs monitor for health disparities as part of healthcare-associated infection (HAI) surveillance through a survey of healthcare epidemiology leaders. Most facilities are not assessing for disparities in HAI rates. Professional society and national guidance should focus on addressing this gap.

Health disparities, defined as “differences in health and well-being outcomes” and health inequities,^1^ when these differences are “avoidable, unfair, and unjust,”^1^ are widespread, including in conditions relevant to healthcare epidemiology and infection prevention teams. There is increasing awareness that systemic and structural factors result in inequitable care based on social determinants of health (SDOH), which are nonmedical factors that influence health outcomes.^2^ For example, inequitable access to resources such as housing and education can result in differential health outcomes.^2^ Limited descriptions exist of how SDOH influence patients’ risks for healthcare-associated infections (HAIs).^3,4^

Health equity and social justice are organizational priorities for the Society of Healthcare Epidemiology of America (SHEA), which has committed to “developing interventions that counter the role of racism, discrimination, and other forms of marginalization that result in inequities of care.”^5^ The Centers for Disease Control (CDC) has declared racism a public health emergency.^6^ However, there are no national standards on whether information related to equity and SDOH should be included in HAI surveillance and how such information should be used.

We sought to determine the proportion of infection prevention (IP) programs that monitor for disparities in their HAI surveillance and what variables are collected. We sought to understand the barriers to monitoring for disparities in HAI surveillance. For programs that currently monitor for disparities, we sought to understand how they use the information.

## Methods

We surveyed members of the Society for Healthcare Epidemiology of America (SHEA) Research Network (SRN),^7^ a consortium of healthcare facilities who collaborate on healthcare epidemiology projects. Respondents were limited to facilities in the United States to account for the contextual nature of health disparities. The survey was administered online via REDCap from October–December 2021 (Supplementary Material online). It was reviewed by the SRN Review Committee and was designated exempt by the Institutional Review Board of the University of Washington. Facilities that do not provide direct patient care were excluded from analyses. Statistical analyses of quantitative data included descriptive statistics and were conducted using Microsoft Excel and Stata version 14 software (StataCorp, College Station, TX). Two team members (C.M. and L.R.) used directed content analysis to analyze the brief free-text responses for concepts corresponding to the Consolidated Framework for Implementation Research (CFIR) domains.^8^ Disagreements were resolved by consensus.

To determine whether facilities were monitoring for disparities or had the data capacity to do so, they were asked “As part of your surveillance program, does your facility collect the following health disparity variables for patients that have HAIs?” with options to select one or more: language for care [referring to the language of healthcare delivery to the patient], race and ethnicity, and insurance status (question 1). These variables were selected because they may be available in many electronic health records (EHRs), and they represent a limited subset of variables that reflect SDOH.

Facilities that selected “yes” to any of the above variables were asked, “Does your facility analyze whether disparities exist (including but not limited to language for care, race and ethnicity, and insurance status) in the rates of any HAIs? This refers to comparing HAI rates by these variables to determine if disparities exist” (question 4). Facilities were asked for which HAIs these variables were being collected and when they began tracking this information (questions 2, 3, 5, and 7). Responses for how information is used (for facilities collecting SDOH variables) and reasons why information is not collected (for facilities not collecting) was elicited via the selection of 1 or more predefined reasons with the option for additional free-text responses (questions 8 and 11).

## Results

The survey was distributed to 68 eligible US-based SRN facilities. 28 (41%) facilities responded and of these, 27 (40%) institutions who provide direct patient care were included. Demographic information was available for 26 (96%) of 27 facilities. Facility types included academic hospitals (15 of 26, 58%), community hospitals (7 of 26, 27%), and other (4 of 26; 16%; including public, federal, and other acute care hospitals). All US regions were represented: Northeast (7 of 26, 27%), South (11 of 6, 42%), Midwest (7 of 26, 27%), West (1of 26, 4%). Total facility bed size ranged from <100 to >1,000 beds. Information about the mix of adult and pediatric beds was available for 25 facilities; 2 (8%) of 25 facilities were predominately pediatric facilities.

**Figure 1.**
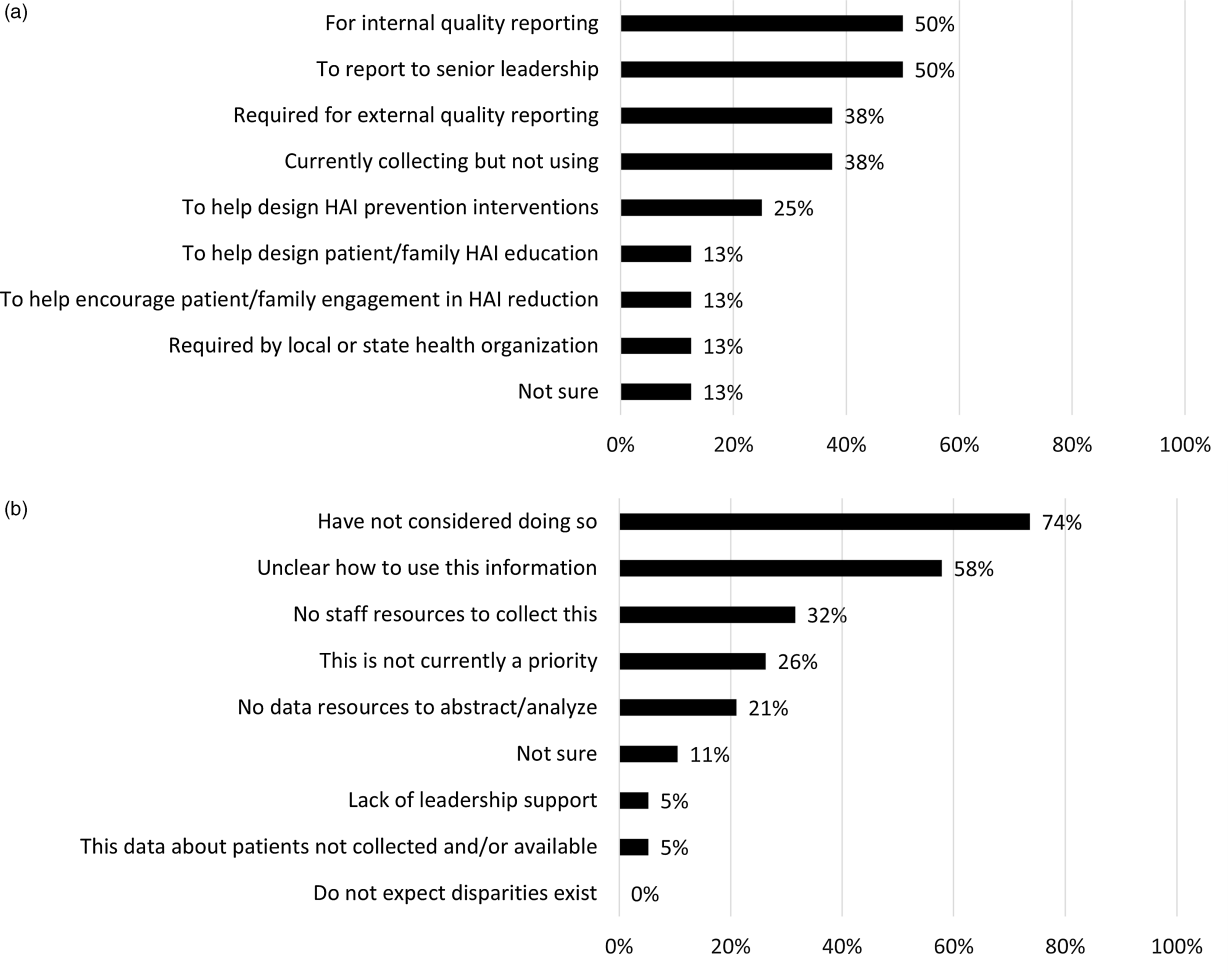
(a) Facility uses for SDOH data for HAI surveillance. Note. Percentage of facilities (of n = 8) currently collecting social determinant of health (SDOH) data for healthcare-associated infection (HAI) surveillance reporting “yes” for each response. (b) Reasons facilities do not collect SDOH data for HAI surveillance. Note. Percentage of 19 facilities not currently collecting social determinant of health (SDOH) data for healthcare-associated infection (HAI) surveillance reporting “yes” for each response. HAI: healthcare-associated infection.

Of 27 facilities, 8 (30%) collected data regarding any variables related to SDOH within the context of HAI surveillance, including language for care (4 of 27, 15%), race and ethnicity (7 of 27, 26%), insurance status (5 of 27, 19%), or other (sex, age group; 3 of 27, 11%). Of those 8 facilities, academic hospitals were overrepresented (7 of 8, 88%). The HAI for which these variables were most commonly collected was central-line–associated bloodstream infection: CLABSI (7 of 8, 88%). Only 3 (11%) of 27 facilities analyzed whether, based on these variables, disparities exist within any HAI rates. Of facilities that collect SDOH variables, 3 (38%) of 8 collect this information but otherwise do not use it; other facilities use this information for purposes ranging from descriptive to action oriented (Fig. 1a). There was a range in how recently facilities began collecting these data: 1 (13%) of 8 began in the last 6 months, 2 (25%) of 8 began in the past 6–12 months, 1 (13%) of 8 began in the past 1–5 years, 2 (25%) of 8 began >5 years ago, and 2 (25%) of 8 were unsure.

The most common reason for not collecting SDOH information within the context of HAI surveillance is that facilities had not considered doing this (14 of 19, 74%) (Fig. 1b), followed by being “unclear about how to use this information” (11 of 19, 58%). Notably, no facilities responded that they “do not expect disparities exist.” Of facilities not yet collecting SDOH variables or monitoring for disparities in HAIs, the majority are interested in doing so: 10 (67%) of 15 were interested and 4 (27%) of 15 were unsure.

Furthermore, 18 respondents provided brief free-text responses. Given the brevity of responses, responses were coded to the level of the CFIR domain and reflected the following domains: innovation (n = 16), outer setting (n = 7), inner setting (n = 2), implementation process (n = 3) (responses could encompass multiple domains). Needs identified from free-text responses and proposed strategies were grouped by CFIR domain (Table 1).

**Table 1.** Identified Needs and Proposed Strategies Related to Monitoring for Disparities in HAI Surveillance

Domain^a^	Specific Identified Needs	Proposed Strategies
Innovation	• Information about HAI disparities and how the intersection of HAI surveillance/prevention and equity is important • Examples of how other facilities have implemented tracking and addressing disparities in HAIs • Standardized process for defining variables, data collection, and analysis • Need for benchmarks	• Provide opportunities for organizations to share experiences with the assessment of disparities in HAIs and examples of how to address identified disparities • Create tool kit with standardized definitions, data fields for collection, and analysis tools • Establish benchmarks • Create a virtual library with examples of how individual facilities have addressed disparities in HAIs in their setting
Inner setting	• Data extraction and analysis tools for electronic medical records • Resources to support time and effort for this surveillance and improvement work	• Toolkits resources should include specific ways to tailor recommendations to a variety of settings
Outer setting	• Professional society position statement on disparities in HAIs • Multicenter data analysis • Data comparison between collecting entities	• Publish SHEA position statement on the importance of health equity within IPC and HAI surveillance with specific recommendations for data collection and analysis • Support organizational advocacy for data fields related to health equity to be required in national surveillance networks • Facilitate working group of interested institutions to provide ongoing support during the implementation process
Implementation process	• Methodology for how to implement assessment for disparities in HAIs including leveraging the electronic medical record	• Create tool kit with steps institutions can follow to implement the assessment for disparities in HAIs

Note. HAI, healthcare-associated infection, SHEA, Society for Healthcare Epidemiology of America; IPC, infection prevention and control.

a From the Center for Implementation Research (CFIR) framework.^8^

## Discussion

Most institutions that participated in this national healthcare facility survey do not monitor for health disparities in HAI rates; however, there is interest in doing so. Very few early-adopter facilities are collecting data or are assessing whether disparities exist through the collection of SDOH variables and designing interventions.

In this survey, we focused on whether facilities are collecting SDOH data, and analyzing these data to assess for disparities in HAI performance. Although collecting and analyzing health disparity data is necessary (to describe the extent of disparities and identify priority areas for intervention), it is only a first step toward providing equitable care.^9^ Subsequent efforts to identify drivers of disparities in HAIs and subsequently mitigate inequities are paramount.^8^

To our knowledge, this is the first large-scale assessment of the current practices of infection prevention programs surrounding the assessment of disparities in HAIs. The strengths of our study include representation of a variety of healthcare facilities throughout the United States.

Feedback revealed several opportunities to support institutions to advance efforts to assess disparities in HAIs. Sharing institutional experiences regarding the identification of disparities in HAIs and subsequent interventions can assist other sites in implementing similar approaches. Multisite collaboration and organizational support to organize and standardize data collection, management, and reporting is necessary. National and professional society guidance should play a key role in standardizing the collection of this information and translating early learnings to identify and subsequently improve equity within HAI performance and prevention strategies. Mandated reporting would increase response rates and allow for targeted interventions.

Our study had several limitations. The response rate was low, which may introduce bias. We only asked about 4 specific SDOH variables that could result in disparities. Many other factors affect how patients receive and experience care, including sexual orientation and gender identity, disability status, housing status, and socioeconomic position; these domains should also be explored for potential disparities in HAIs. Our survey methodology and brief free-text responses did not allow us to explore responses in greater detail. Interviews or focus groups could facilitate greater understanding of certain aspects such as why some facilities do not consider the assessment of disparities in HAIs to be a priority.

This exploratory survey highlights that most facilities are not assessing for health disparities in HAI rates and underscores that many opportunities exist to advance this work. National or professional society guidance on how to assess for health disparities in HAIs and how to use this information to improve prevention strategies could serve as an essential step in the process of mitigating health inequities in healthcare-associated infections.

## Supplementary material

To view supplementary material for this article, please visit https://doi.org/10.1017/ice.2023.181
